# Expectations and limitations of biotechnology in the perspective of 2050

**DOI:** 10.5114/bta/203189

**Published:** 2025-03-31

**Authors:** Tomasz Twardowski

**Affiliations:** Institute of Bioorganic Chemistry PAS; Former Editor-in-Chief “BioTechnologia”; Honorary Chairman Biotechnology Committee PAS

The first quarter of the 21^st^ century has already passed. The perspective of 2050 draws closer, bringing various pressing issues with serious consequences: the global population will likely reach 9 billion, there will not be enough land or fresh water for agriculture, and environmental pollution and energy shortages will persist. Drought, heat, and global warming are and will continue to be critically important for agricultural biotechnology and sustainable food and feed production. The same concerns apply to medical science and healthcare: new diagnostic tools and breakthrough drugs are urgently needed (e.g., personalized medicine). Above all, the rapid development of bioinformatics and artificial intelligence stands out as the most significant factor for future progress.

Naturally, the question arises: what factors limit the advancement of scientific research and the transfer of innovative technologies from the research laboratory to industry? In my opinion, two fundamental, closely related factors are at play: financial investment and social acceptance of innovative solutions. A seemingly simple solution—social education—requires substantial time and financial resources. While these two factors are critical, several other essential conditions influence the smooth development of biotechnology and the translation of scientific achievements into everyday life. Contrary to appearances, these are not purely scientific or technical conditions. Another fundamental factor is legal norms, including intellectual property rights. Legal regulations are directly dependent on public opinion (as elected parliaments typically adopt legal norms), but international agreements in this area are also difficult to achieve. These challenges are well illustrated by the legal situation regarding NGT-1 and NGT-2 (new genomic techniques) and, consequently, the issue of patenting innovative solutions in agrobiotechnology. These problems are clearly described in EU-SAGE’s materials [EU SAGE = European Sustainable Agriculture through Genome Editing, see https://www.eu-sage.eu]. For a contrary view, see the statement by the opposition to modern biotech: European small-scale farmers call on Member States to not support Poland’s proposal on NGTs https://www.eurovia.org/news/statementeuropean-small-scale-farmers-call-on-member-states-to-not-support-polands-proposal-on-ngts/.

Transferring academic knowledge to industry, or implementing innovative concepts into the bioeconomy, is a difficult task. However, an even greater challenge is outlining the direction of development and prospective research in the near and distant future. Managing and guiding the future development of the bioeconomy (and, subsequently, the circular bioeconomy) is closely related to social changes and the role of various stakeholders, as reflected in the actions and views of policymakers. In the long run, it is necessary to create not only new implementation concepts, such as land and freshwater use, but also advances in medical technologies and products, such as innovative drugs, non-conventional vaccines, and diagnostics based on hormone or sequence analysis. A perfect example illustrating these issues is the recent COVID-19 pandemic. The rapid spread of the virus on a global scale, followed by molecular diagnostics and vaccine development in an extremely short period, is an impressive example of academic knowledge transferring into widespread medical use. Similar prospects and limitations apply to environmental cleaning and renewable energy acquisition.

All these issues have both promising and challenging examples. A recent illustration of progress in understanding and acceptance comes from Ethiopia. According to Crop Biotech News (March 5, 2025), Ethiopia has approved the commercial release of genetically modified (GM) insect-protected maize. Additionally, the Ethiopian Government has authorized the commercialization of GM cotton varieties, a significant development for Ethiopian society.

As previously mentioned, bioinformatics and artificial intelligence are the common denominators for all achievements and further perspectives. In particular, their role in interpreting the multifaceted issues of contemporary and future bioeconomy, determining development directions, and selecting priority goals cannot be overstated. At the same time, these ultramodern fields of science and technology highlight how a small percentage of both society and the academic world truly understands and utilizes these advancements. This is another very strong argument in support of the need for education—not only for scientific staff but also for the general public, extending beyond biotechnology alone. In addressing these challenges, a seemingly simple but essential factor is the terminology used, which should be understandable to consumers of scientific achievements.

The perspective of 2050 is still 25 years away, allowing us to forecast based on the past 25 years. The balance and effects of the first quarter of the 21^st^ century yield mixed conclusions. We have witnessed great successes: innovative diagnostic techniques and drugs, bioinformatic analyses, and genome sequencing of numerous organisms. We can now modify genomes to produce genetically modified organisms (GMOs) with desired properties. However, paradoxically, in wealthy countries, antivaccine movements and GM food rejection persist, while nearly a billion people in poor regions suffer from hunger. These stark contradictions can be addressed through universal education and the application of innovative science for the benefit of all. This is the challenge ahead of us. However, none of these issues are new; as was said in ancient Rome, “Nihil novi sub sole”.

All these factors significantly influence the transformation of our economy toward a bioeconomy and, in the long term, a circular bioeconomy based on gene regulation at the molecular level. This raises the question: what will be the directions of “bio” development shortly? Certainly, the circular bioeconomy will be a key focus. However, special importance should be placed on bioinformatics and artificial intelligence, biomaterials and bioenergetics, environmental biotechnology, and medical technology advancements (e.g., nanomedicine, diagnostics, biomedicine). Additionally, new directions will likely emerge that we cannot yet anticipate. Personally, I am very confident that the bioeconomy is the future of the world.

The *BioTechnologia* journal and its editorial team will delve into these groundbreaking discoveries, aiming to contribute to the global development of biotechnology in both academic research and industry by promoting extensive information sharing and expertise exchange.

In conclusion, I cordially invite you to explore the fascinating and valuable articles in the current and future issues of the quarterly.

I wish you an enjoyable read.

